# Heterometal Dopant Changes the Mechanism of Proton-Coupled Electron Transfer at the Polyoxovanadate-Alkoxide Surface

**DOI:** 10.1021/jacs.3c14054

**Published:** 2024-01-19

**Authors:** Shannon E. Cooney, M. Rebecca A. Walls, Eric Schreiber, William W. Brennessel, Ellen M. Matson

**Affiliations:** Department of Chemistry, University of Rochester, Rochester, New York 14627, United States

## Abstract

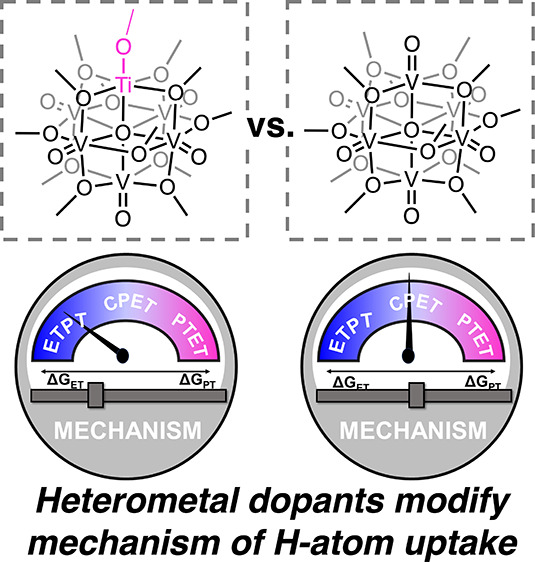

The transfer of two H-atom equivalents to the titanium-doped polyoxovanadate-alkoxide, [TiV_5_O_6_(OCH_3_)_13_], results in the formation of a V(III)–OH_2_ site at the surface of the assembly. Incorporation of the group (IV) metal ion results in a weakening of the O–H bonds of [TiV_5_O_5_(OH_2_)(OCH_3_)_13_] in comparison to its homometallic congener, [V_6_O_6_(OH_2_)(OCH_3_)_12_], resembling more closely the thermodynamics reported for the one-electron reduced derivative, [V_6_O_6_(OH_2_)(OCH_3_)_12_]^1–^. An analysis of early time points of the reaction of [TiV_5_O_6_(OCH_3_)_13_] and 5,10-dihydrophenazine reveals the formation of an oxidized substrate, suggesting that proton-coupled electron transfer proceeds via initial electron transfer from substrate to cluster prior to proton transfer. These results demonstrate the profound influence of heterometal dopants on the mechanism of PCET with respect to the surface of the assembly.

Proton-coupled electron transfer (PCET) is a fundamental reaction in energy storage and conversion processes.^1−3^ The transfer of proton and electron pairs can proceed through multiple mechanisms, either the sequential movement of electrons and protons (e.g., electron transfer followed by proton transfer, ET/PT) or the synchronous delivery of an H-atom equivalent (e.g., concerted proton electron transfer, CPET).^2^ Understanding factors that influence the mechanism of PCET is important, as the pathway of delivery of H-atom equivalents has been shown to dictate product selectivity in small-molecule activation reactions.^4,5^

In the past decade, PCET has emerged as a model for understanding charge transfer reactions at the surface of reducible metal oxides.^6,7^ Mayer and co-workers have provided evidence for the formation of reactive H-atom equivalents at the surface of reduced metal oxide nanoparticles that are capable of hydrogenating small-molecule substrates (e.g., quinones, TEMPO).^8^ Subsequent work has established structure–function relationships that dictate the thermodynamics of PCET at the surface of metal oxides nanocrystals. For example, the size, degree of reduction, and coverage of CeO_*x*_ nanoparticles are shown to influence the bond dissociation free energy of surface hydroxide groups, BDFE(O–H).^9,10^ However, little is understood about the relationship between the composition and the mechanism of interfacial PCET reactions.^7^

Our research team is investigating H-atom uptake and transfer in a family of polyoxovanadate-alkoxide (POV-alkoxide) clusters (Figure 1).^11−16^ We view these hexavanadate assemblies as molecular models for the surface of colloidal metal oxide nanoparticles. POV-alkoxides are composed of alternating terminal and bridging oxide sites, broadly resembling the surfaces of extended solids.^17−20^ Reduced forms of these clusters possess Robin and Day Class II delocalized electronic structures that mimic electronic communication invoked in reducible metal oxide materials.^21,22^ However, unlike their bulk congeners, the monodisperity of molecular POV-alkoxides provides access to atomically resolved depictions of surface reactivity.

**Figure 1:**
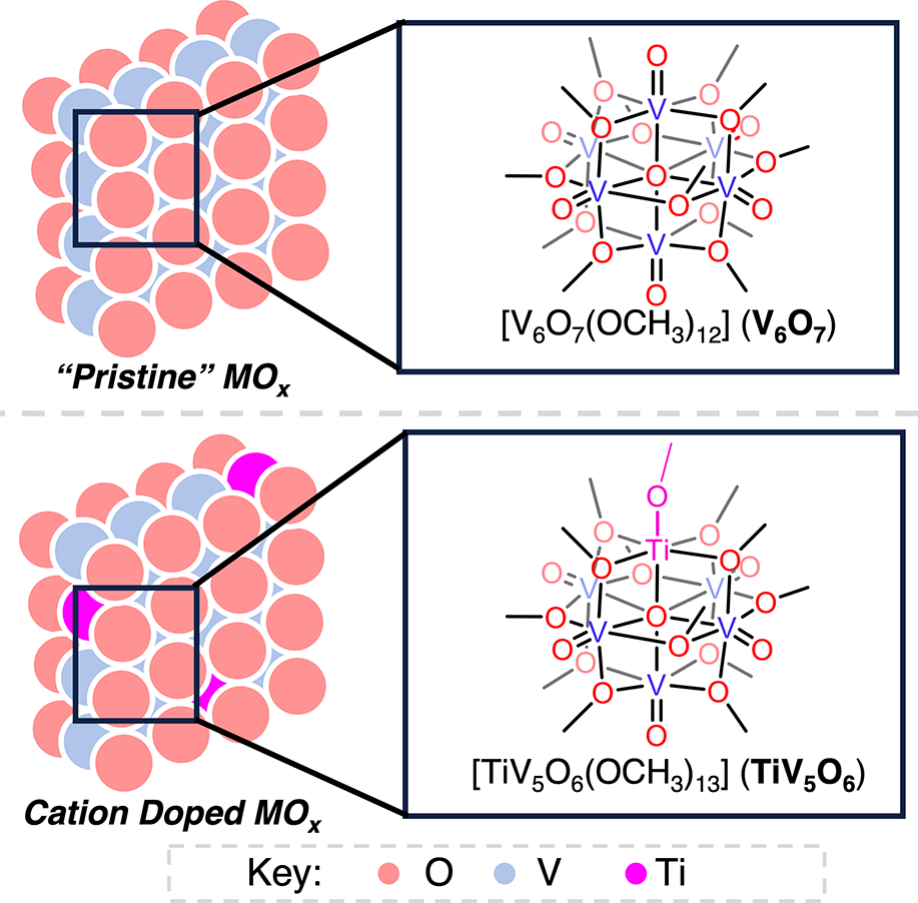
Homo- and heterometallic polyoxovanadate-alkoxide clusters serve as models for the surface reactivity of plenary and heterometal-doped reducible metal oxides.

To this end, we became interested in leveraging our expertise in the manipulation of the metal composition of POV-alkoxides to model the impact of heterometal dopants on H-atom uptake at metal oxide surfaces.^23−28^ Heterometal dopants have been shown to impact lattice geometries and electronic structures of metal oxides, dictating the reactivity at the solution–solid interface.^29^ Specifically, these dopants cause geometric strain across the lattice, activating the neighboring oxide moieties. Similar structural distortions of POV-alkoxides are observed upon installation of a heterometal dopant; installation of a titanium center results in the elongation of V–O bonds of vanadium centers adjacent to the dopant.^25^ We thus hypothesized that the thermodynamics and kinetics of H-atom uptake at the cluster surface would be influenced.

Initial experiments probed the reduction of the Ti-functionalized POV-alkoxide cluster, [^n^Bu_4_N][TiV_5_O_6_(OCH_3_)_13_] (TiV_5_O_6_^1–^). Exposure of TiV_5_O_6_^1–^ to 5,10-dihydrophenazine (H_2_Phen) results in no consumption of the starting material, despite prolonged reaction times and elevated temperatures (Figure S1). This result is unsurprising, given that all vanadium centers in TiV_5_O_6_^1–^ are in the 4+ oxidation state.^25,30^ Previous work has established that O-atom defect formation requires a mixed-valent POV-alkoxide starting material (i.e., a cluster with at least one vanadium(V) center).^31,32^ As such, our attention shifted to the reactivity of the 1e^–^ oxidized assembly, [TiV_5_O_6_(OCH_3_)_13_] (TiV_5_O_6_).

While we have reported previously the *in situ* oxidation of TiV_5_O_6_^1–^,^30^ the isolation of TiV_5_O_6_ has not been described. Oxidation of TiV_5_O_6_^1–^ was accomplished via addition of 1 equivalent of silver trifluoromethylsulfonate (AgOTf, Scheme 1). Following workup, the analysis of the product by ^1^H NMR spectroscopy revealed four paramagnetically broadened and shifted resonances, distinct from the spectrum of the starting material (Figure S2). The electronic absorption spectrum (EAS) of TiV_5_O_6_ confirms the oxidation of a V(IV) center; intervalence charge transfer (IVCT) bands are observed at 384 nm (ε = 4185 M^–1^ cm^–1^) and 1095 nm (ε = 530 M^–1^ cm^–1^) (Figure 2). Additional evidence for cluster oxidation localized at vanadium is observed in structural data obtained through single crystal X-ray diffraction (Figure S4 and Tables S1–S3); bond valence sum calculations support the assignment of the oxidation state distribution of TiV_5_O_6_ as Ti^IV^V^IV^_4_V^V^.

**Scheme 1:**
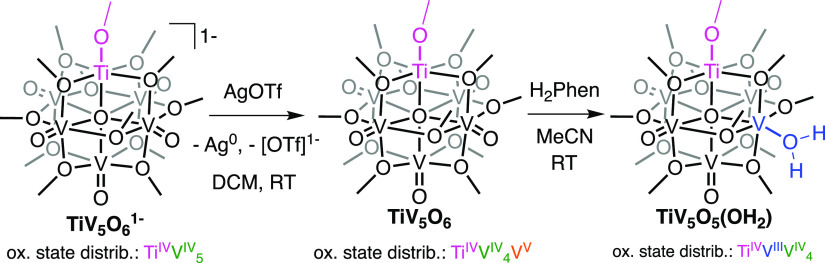
Synthesis of TiV_5_O_6_ and TiV_5_O_5_(OH_2_)

**Figure 2:**
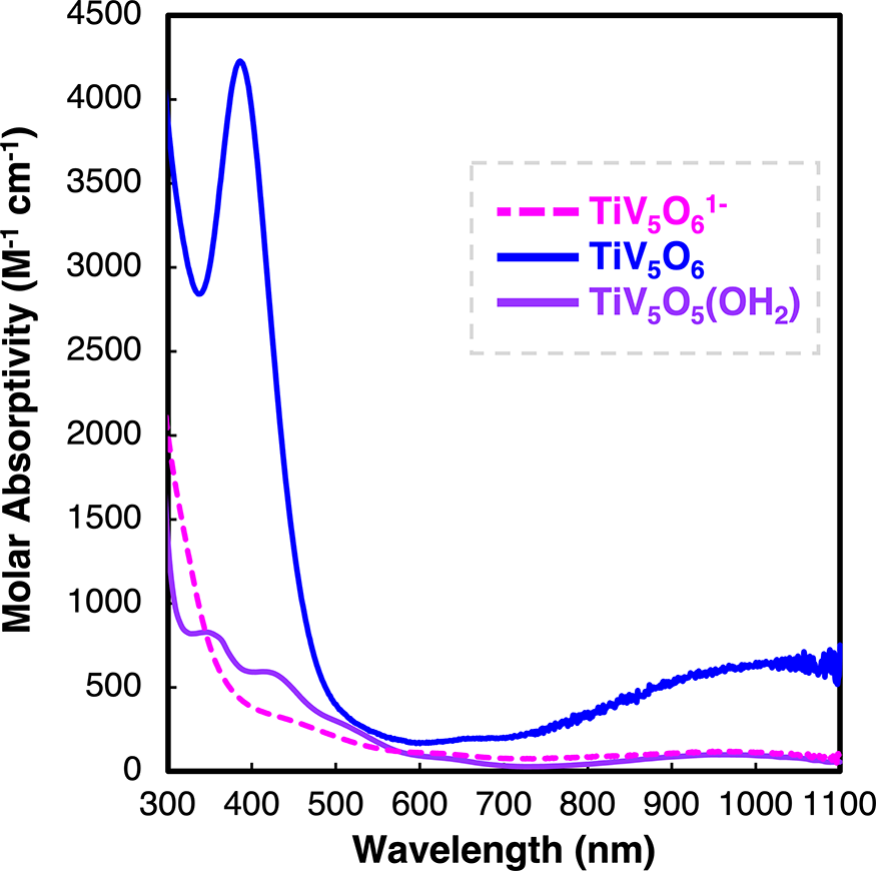
Electronic absorption spectra of TiV_5_O_6_^1–^ (0.74 mM), TiV_5_O_6_ (0.28 mM), and TiV_5_O_5_(OH_2_) (0.81 mM) collected in MeCN at room temperature (21 °C).

The addition of 1 equivalent of H_2_Phen to TiV_5_O_6_ results in an immediate color change from green to orange (Scheme 1). An analysis of the crude product by ^1^H NMR spectroscopy reveals the formation of phenazine and water, indicating successful delivery of 2 H-atom equivalents to the surface of the cluster to generate [TiV_5_O_6_(OCH_3_)_13_] (TiV_5_O_5_(OH_2_); Figure S5). The paramagnetic region of the ^1^H NMR spectrum of TiV_5_O_5_(OH_2_) is more complicated than that of the starting material (Figure S6). The increase in the number of paramagnetically broadened and shifted signals (12 vs 4) indicates a reduction in symmetry of the cluster following H-atom uptake (C_4v_ → C_s_). This observation is consistent with the reduction of a vanadyl moiety (V^V^=O → V^III^–OH_2_) adjacent to the titanium dopant. Indeed, O-atom defect formation at the vanadium center *trans* to the titanium dopant would result in the retention of C_4v_ symmetry of the parent cluster, resulting in only four signals for the product. Additionally, multiple signals shifted upfield from the diamagnetic region are observed in the spectrum of TiV_5_O_5_(OH_2_). Resonances with similar chemical shifts have been observed following the formation of an O-atom defect at the surface of POV-alkoxides.^14,31−34^

Further evidence of O-atom defect formation was noted in the EAS of the product (Figure 2); loss of the V^IV^ → V^V^ IVCT bands is consistent with reduction of the V(V) to V(III) upon H-atom uptake. Additional new transitions are observed at 356 (ε = 810 M^–1^ cm^–1^), 428 (ε = 580 M^–1^ cm^–1^), and 524 nm (ε = 240 M^–1^ cm^–1^). These absorptions of TiV_5_O_5_(OH_2_) resemble those reported previously for O-atom-deficient POV-alkoxide clusters^31,32^ and are assigned as d–d excitations of the three chemically distinct V(IV) ions of the product (Figure S7). The fact that three transitions are observed offers further support for defect formation at a *cis* vanadyl ion; in a cluster with a *trans* defect, all V(IV) ions would be positioned in the equatorial plane of the cluster, resulting in chemical equivalency (i.e., one anticipated d–d transition in the absorption spectrum). The bulk purity of TiV_5_O_5_(OH_2_) was confirmed through elemental analysis with only one O-atom defect present on the cluster.

The observation of the formation of an O-atom defect at a vanadium center positioned *cis* to the titanium dopant is significant. Theoretical investigations interrogating defect formation in doped metal oxides reveal preferential reduction adjacent to the heterometal.^35−38^ This observation is justified by the fact that the heterometal weakens adjacent metal oxygen bonds. It is important to recall that in the case of these low-valent POV-alkoxide clusters, bridging sites are saturated with alkoxide ligands, rendering them inert to H-atom-transfer reactions. H-atom uptake at TiV_5_O_6_ occurs at the surface oxide ligand positioned closest to the heterometal, resulting in site selectivity of O-atom defect formation that is similar to that observed in doped transition-metal oxides.

We next evaluated the effect of the Ti dopant on the thermodynamics of H-atom uptake via quantification of BDFE(O–H)_avg_ of the aquo ligand of TiV_5_O_5_(OH_2_). The use of a more mild reductant, hydrazobenzene (H_2_Azo, BDFE(N–H)_avg_^THF^ = 60.4 kcal/mol^3,39^), results in partial conversion to products, establishing an equilibrium at which the affinities of H atoms for both substrate and cluster are identical. Under these conditions, the effective BDFE(O–H)_avg_ of the cluster can be quantified (details in the SI).^9,14,16,34^ The BDFE(O–H)_avg_ for TiV_5_O_5_(OH_2_) was determined to be 60.1 ± 0.1 kcal/mol (Figure S8, Table S4).

A comparison of the BDFE(O–H)_avg_ value of TiV_5_O_5_(OH_2_) to that of its homometallic congener, [V_6_O_6_(OH_2_)(OCH_3_)_12_] (V_6_O_6_(OH_2_), BDFE(O–H)_avg_ = 62.3 ± 0.1 kcal/mol) reveals weakening of the O–H bonds of the surface aquo ligand.^16^ The experimental BDFE(O–H)_avg_ values of TiV_5_O_5_(OH_2_) are statistically equivalent to that of the one-electron reduced assembly, [V_6_O_7_(OCH_3_)_12_]^1–^ (V_6_O_6_(OH_2_)^1–^; BDFE(O–H)_avg_ = 59.9 ± 0.1 kcal/mol).^34^ Trends in BDFE(O–H)_avg_ values across the three POV-alkoxide clusters can be justified by taking into consideration the Lewis acidity of the metal ions composing the cluster core. In this scenario, we consider the three clusters with a generic formula of MV^III^V^IV^_4_, where M = Ti(IV) (TiV_5_O_5_(OH_2_)), V(IV) (V_6_O_6_(OH_2_)^1–^), or V(V) (V_6_O_6_(OH_2_)); this treatment allows for the assessment of the impact of the single unique metal contained within the Lindqvist core. A comparison of the Lewis acid strength of the ions reveals similar acidities for V(IV) (0.71) and Ti(IV) (0.67), whereas V(V) is more electropositive (1.2).^40,41^ The correlation between Lewis acidities of the unique metal center and observed BDFE(O–H)_avg_ values indicates that the collective electron affinity of metal ions within the assembly dictates the strength of surface O–H bonds in reduced materials.

To explore the mechanism of PCET in TiV_5_O_6_, kinetic investigations were performed. The addition of excess reductant to TiV_5_O_6_ in acetonitrile (MeCN) results in immediate quenching of the IVCT band located at 1050 nm at −35 °C (60% complete at the initial time point, *t*_i_; Figure S9). Closer inspection of the full EAS at low temperature reveals the rapid formation of an intermediate, with intense transitions located at 400–500 and 550–750 nm which disappear over the course of 1 h (Figure 3). These spectral features match the EAS for the one-electron oxidized form of the substrate, H_2_Phen^**·**+^,^42^ unique from H_2_Phen and Phen (Figure S10), suggesting that PCET to the surface of the Ti-doped POV-alkoxide proceeds through an initial electron transfer (ET) step, followed by a rate-determining proton transfer (PT; Figure 4). In contrast, the addition of excess H_2_Phen to V_6_O_7_^1–^ at −35 °C results in the direct, gradual conversion to V_6_O_6_^1–^ over the course of 2.5 h (Figure 3), consistent with the reported mechanism of concerted PCET for H-atom uptake.

**Figure 3:**
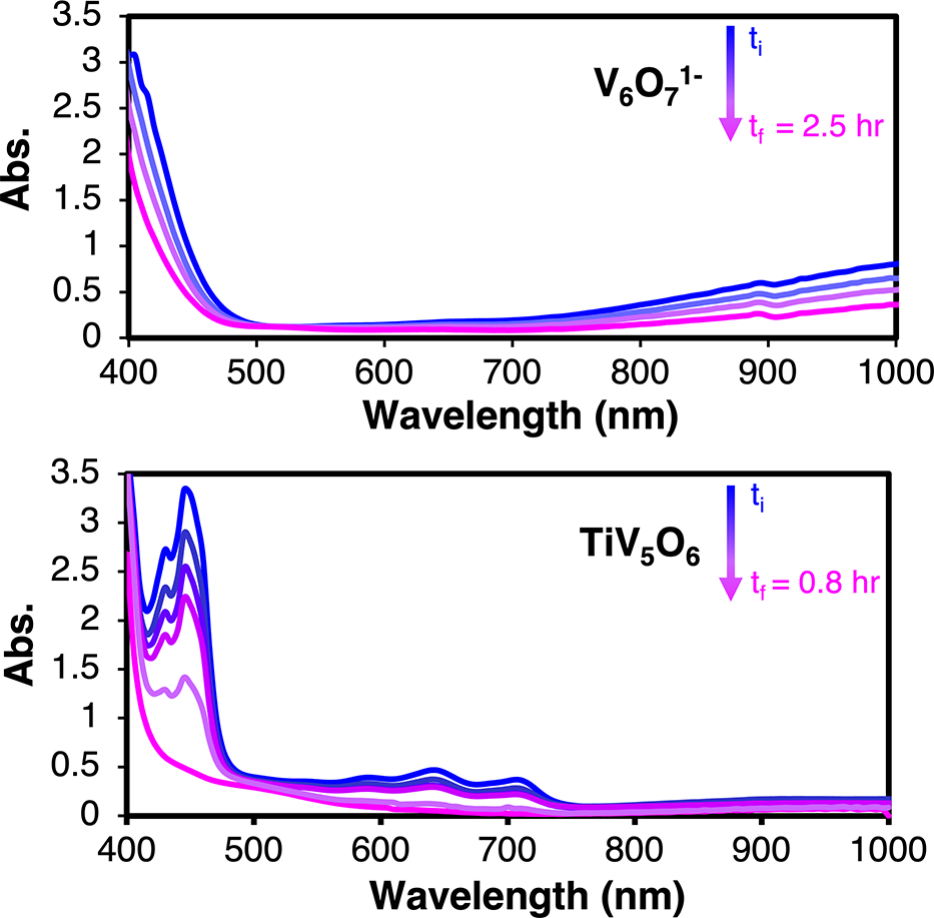
Scanning kinetics of V_6_O_7_^1^^–^ (0.75 mM) + 10 equiv of H_2_Phen (7.5 mM) (top) and TiV_5_O_6_ (0.75 mM) + 10 equiv of H_2_Phen (7.5 mM) (bottom) at −35 °C in MeCN.

**Figure 4:**
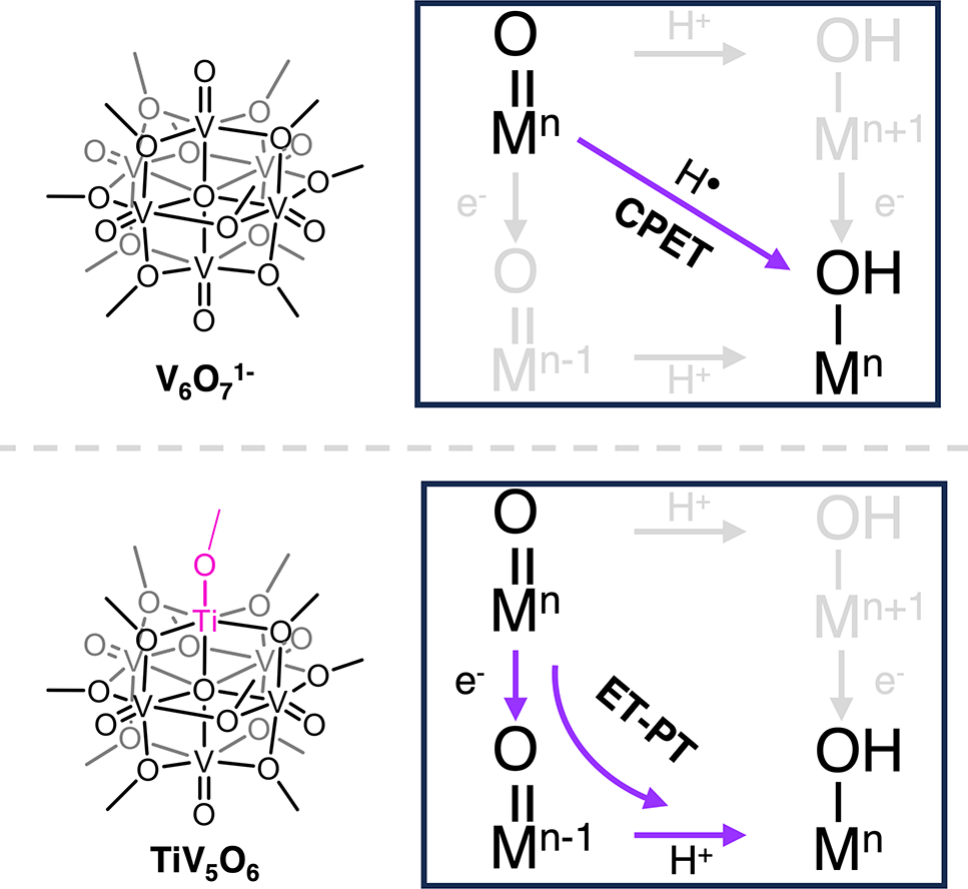
Distinct mechanisms of H-atom uptake at POV-alkoxide surfaces are dependent on the composition of transition metals within the Lindqvist core.

In an attempt to resolve the rates of the ET and PT steps of H-atom uptake at TiV_5_O_6_, we repeated kinetic analyses in tetrahydrofuran (THF). This solvent was selected for its lower freezing point and decreased dipole moment, which are factors that slow ET.^43^ While the initial ET from H_2_Phen to TiV_5_O_6_ in THF remains too rapid for initial rates to be reliably obtained (∼13% IVCT quenching observed by *t*_i_; Figure S11), a lower bound for *k*_ET_ was found (*k*_obsET_ > 0.17 s^–1^). Coincident with the loss of the IVCT band at 1050 nm, growth of the transitions assigned to the intermediate H_2_Phen^**·**+^ were observed. Consumption of H_2_Phen^**·**+^ was monitored at 645 nm to obtain the rate for the subsequent PT step (*k*_obsPT_ = (9 ± 1) × 10^–4^ s^–1^, *k*_PT_ = 0.028 ± 0.005 M^–1^ s^–1^, Figure S12). PT is the rate-determining step for PCET to TiV_5_O_6_, providing a quantitative measure of the rate of H-atom uptake at the cation-doped cluster surface. A comparison of this value to the rate constant for *k*_PCET_ measured for V_6_O_7_^1–^ in THF (*k*_PCET_ = (3.5 ± 0.1) × 10^–4^ M^–1^ s^–1^ at −50 °C, Figures S13 and S14) reveals that H-atom uptake at TiV_5_O_6_ is accelerated.

The observed discrepancies in the mechanism of PCET at the two POV-alkoxide surfaces are justified through a comparison of thermodynamic driving forces for electron transfer between H_2_Phen and V_6_O_7_^1–^ and TiV_5_O_6_. Incorporation of the heterometal dopant into the Lindqvist core results in a substantially anodic shift in its reduction potential versus its homometallic congener (*E*_1/2_^red^ = −0.29 V (TiV_5_O_6_), −0.78 V (V_6_O_7_^1–^) vs Fc^+/0^).^25^ The more facile reducibility of the assembly enables spontaneous electron transfer from H_2_Phen (*E*_1/2_^ox^ = −0.33 V vs Fc^+/0^, Δ*G*_ET_ = −0.92 kcal mol^–1^).^12^ In contrast, the more cathodic reduction potential of V_6_O_7_^1–^ renders H_2_Phen incapable of reducing the assembly via ET (Δ*G*_ET_ = +10.38 kcal mol^–1^).^44^

Strategies for modifying PCET mechanisms have recently been credited to three factors: balancing of Δ*G*_ET_ and Δ*G*_PT_, steric hindrance of the proton-transfer coordinate, and isotope substitution.^45^ In this study, we demonstrate that heterometal dopants enable a change in mechanism of H-atom uptake from concerted (CPET) to stepwise (ET-PT) at the surface of a nanoscopic metal oxide assembly by minimizing Δ*G*_ET_. Modifying the mechanism of H-atom uptake at the surface of the POV-alkoxide results in a drastic acceleration of defect formation in the case of the doped assembly in comparison to its homometallic analogue, despite similar thermodynamic driving forces for the two reactions. This molecular modification differs from approaches reported previously, which focus on the impact of modifying reaction conditions (i.e., p*K*_a_ of organic acids, etc).^46−49^ Additional mechanistic investigations detailing the reactivity of heterometal-doped POV-alkoxide clusters that will provide additional predictive capabilities for the behavior of PCET reactivity at the surface metal oxides are underway.

## Supporting Information Available

Synthesis and additional experimental details and spectroscopic data supporting the formation of TiV_5_O_6_ and TiV_5_O_5_(OH_2_) (PDF)
