# Supplementary material for: Heterometal Dopant Changes the Mechanism of Proton-Coupled Electron Transfer at the Polyoxovanadate-Alkoxide Surface
Source: J Am Chem Soc. 2024 Jan 19;146(4):2364–9. doi: 10.1021/jacs.3c14054 (PMC10835708; doi:10.1021/jacs.3c14054)
Supplement: Supplementary file 1 — ja3c14054_si_001.pdf [file ja3c14054_si_001.pdf]

ELECTRONIC SUPPORTING INFORMATION FILE

Heterometal Dopant Changes the Mechanism of Proton-Coupled Electron Transfer at the Polyoxovanadate-Alkoxide Surface

Shannon E. Cooney, M. Rebecca A. Walls, Eric Schreiber, William W. Brennessel, Ellen M. Matson*

Department of Chemistry, University of Rochester, Rochester, New York 14627, USA

Supporting Information Table of Contents

Experimental S2

Figure S1. ^{1}H NMR spectra of $\mathbf{TiV_5O_6^{1-}}$ + 1 equiv H_2Phen in MeCN-d_3 at 21 °C and after heating. S6

Figure S2. ^{1}H NMR spectra of $\mathbf{TiV_5O_6^{1-}}$ and $\mathbf{TiV_5O_6}$ in MeCN-d_3 at 21 °C. S7

Figure S3. Infrared spectra of $\mathbf{TiV_5O_6}$ and $\mathbf{TiV_5O_5(OH_2)}$ S7

Figure S4. Crystal Structure of $\mathbf{TiV_5O_6}$ S8

Table S1. Crystal data and structure refinement for $\mathbf{TiV_5O_6}$ S8

Table S2. Structural parameters of $\mathbf{TiV_5O_6}$ and $\mathbf{TiV_5O_6^{1-}}$ S9

Table S3. Bond valence sum calculations for vanadium atoms in $\mathbf{TiV_5O_6}$ S9

Figure S5. ^{1}H NMR of $\mathbf{TiV_5O_6}$ + 1 equiv H_2Phen in MeCN-d_3 at 21 °C S10

Figure S6. ^{1}H NMR of $\mathbf{TiV_5O_6}$ and $\mathbf{TiV_5O_5(OH_2)}$ in THF-d_8 at 21 °C S11

Figure S7. Illustration of $\mathbf{TiV_5O_5(OH_2)}$ to delineate chemically unique V(IV) centers S11

Figure S8. ^{1}H NMR of $\mathbf{TiV_5O_6}$ + 1 equiv H_2Azo in THF-d_8 ....S12

Table S4. BDFE(O-H)$_{adj}$ calculated from equilibrium reactions of $\mathbf{TiV_5O_6}$ with H_2Azo S12

Figure S9. EAS of $\mathbf{TiV_5O_6}$ +10 equiv H_2Phen in MeCN over time in the near IR, -35 °C........S13

Figure S10. EAS of H_2Phen and Phen, collected in MeCN at 21 ºC........S13

Figure S11. Determining a lower bound of ET (k_{obsET}) to $\mathbf{TiV_5O_6}$ and formation of $H_2Phen^{\cdot+}$ in THF ...S14

Figure S12. Determining PT (k_{obsPT}) to $\mathbf{TiV_5O_6^{1-}}$ and loss of $H_2Phen^{\cdot+}$ in THF S14

Figure S13. Kinetic traces for the reaction of $\mathbf{V_6O_7^{1-}}$ + XS H_2Phen in THF at variable temperatures S15

Figure S14. Eyring Parameters for the reaction of $\mathbf{V_6O_7^{1-}}$ + H_2Phen in THF........S16

References.. S17

General Considerations.

All manipulations were carried out in the absence of water and oxygen using standard Schlenk techniques or in a UniLab MBraun inert atmosphere drybox under a dinitrogen atmosphere. All glassware was oven-dried for a minimum of 4 h and cooled in an evacuated antechamber prior to use in the drybox. Solvents were dried and deoxygenated on a glass contour system (Pure Process Technology, LLC) and stored over 4 Å molecular sieves purchased from Fisher Scientific and activated prior to use. Tetrabutylammonium borohydride ([^{n}Bu$_4$N][BH$_4$]), titanium (IV) isopropoxide, silver trifluoromethanesulfonate, 5,10-Phenazine (Phen), and hydrazobenzene (H_2Azo), purchased from Sigma-Aldrich and used as received. $[V_6O_7(OCH_3)_{12}]^{1-}$, 5,10-dihydrophenazine (H_2Phen), $VO(OCH_3)_3$, were generated according to literature precedent.[1-3]

^{1}H NMR spectra were recorded at 500 MHz on a Bruker DPX-500 MHz spectrometer locked on the signal of deuterated solvents. All chemical shifts were reported relative to the peak of residual H signal in deuterated solvents. THF-d_8 and MeCN-d_3 were purchased from Cambridge Isotope Laboratories and stored in the drybox over activated 4 Å molecular sieves. Infrared (FT-IR, ATR) spectra of complexes were recorded on a Perkin Elmer Spectrum 3 Fourier Transform Infrared Spectrophotometer and are reported in wavenumbers (cm^{-1}). Electronic absorption measurements were recorded at room temperature in anhydrous acetonitrile in a sealed 1 cm quartz cuvette with an Agilent Cary 60 UV-Vis spectrophotometer or an Agilent Cary 3500 spectrophotometer. Kinetic experiments were carried out on an Agilient Cary 60 UV-Vis spectrophotometer held at desired temperatures using an Unisoku CoolSpek UV cryostat, as well as an Agilent Cary 3500 UV-Vis spectrophotometer held at desired temperatures with an integrated Peltier temperature control system.

A single crystal of **$TiV_5O_6^0$** was placed onto a thin glass optical fiber and mounted on a Rigaku XtaLAB Synergy-S Dualflex diffractometer equipped with a HyPix-6000HE HPC area detector for data collection at 100.00(10) K. A preliminary set of cell constants and an orientation matrix were calculated from a small sampling of reflections.[4] A short pre-experiment was run, from which an optimal data collection strategy was determined. The full data collection was carried out using a PhotonJet (Cu) X-ray source with frame times of 2.15 and 8.59 seconds and a detector distance of 34 mm. Series of frames were collected in 0.50° steps in ω at different 2θ, κ, and ϕ settings. After the intensity data were corrected for absorption, the final cell constants were calculated from the xyz centroids of 25572 strong reflections from the actual data collection after integration.[5] See Table S1 for additional crystal and refinement information. The structure was solved using SHELXT[4] and refined using SHELXL.[6] The space group $P42_12$ was determined based on intensity statistics. All hydrogen atoms were placed in ideal positions and refined as riding atoms with relative isotropic displacement parameters. The crystal was an inversion twin whose component mass ratio refined to 0.64:0.36. The final full matrix least squares refinement converged to $R1 = 0.0480$ (F-, $I > 2s(I)$) and $wR^2 = 0.1365$ (F^2, all data).

Elemental analysis was performed on a PerkinElmer 2400 Series II Analyzer, at the CENTC Elemental Analysis Facility, University of Rochester.

Synthesis of [^{n}Bu$_4$N][TiV$_5$O$_6$(OCH$_3$)$_{13}$] (**$TiV_5O_6^{1-}$**). The preparation of the monoanionic TiPOV-alkoxide cluster was accomplished via modified synthetic procedure.[7] A In a glovebox, $VO(OCH_3)_3$ (0.450 g, 2.812 mmol) and [^{n}Bu$_4$N][BH$_4$](0.146 g, 0.568 mmol) were added as solids into two 50 mL Teflon-lined autoclave reactors. 25 mL of MeOH was added to each mixture and immediately turned blue-green as gas evolved. Ti(O^{i}Pr)$_4$ (0.160 g, 0.563 mmol) was added as a liquid by mass to the reaction mixtures. The autoclave reactors were sealed, and the mixtures heated in an oven at 125 °C for 24 hours. The autoclave reactors were allowed to cool to room temperature, then opened outside of the glovebox. A brown solution resulted with teal precipitate. The solid was gravity filtered off and the dark brown solutions were combined, and solvent was removed under vacuum to give a red-brown solid. The solid was washed with toluene (5 x 100 mL) until the solvent ran clear and extracted in 20 mL THF until the brown solid was dissolved. Volatiles were removed under vacuum to yield [^{n}Bu$_4$N][TiV$_5$O$_6$(OCH$_3$)$_{13}$] as a brown solid (1.141 g, 1.093 mmol,

97%). Characterization of the product, [^{n}Bu$_4$N][TiV$_5$O$_6$(OCH$_3$)$_{13}$], matched that previously reported by our research group.[7]

Synthesis of [TiV$_5$O$_6$(OCH$_3$)$_{13}$] (**TiV$_5$O$_6$**). In a glovebox, A 20 mL scintillation vial was charged with **TiV$_5$O$_6$$^{1-}$** (0.119 g, 0.11 mmol), AgOTf (0.032 g, 0.12 mmol), and 10 mL of dichloromethane. The color immediately changed from brown to green with a grey precipitate. The reaction stirred for 30 min to ensure completion. The precipitate was filtered off over celite and the solvent was removed under reduced pressure. The green solid was extracted with hot diethyl ether, 50 °C, (3 x 10 mL) and passed over a silica plug. The solid was dried from the diethyl ether to reveal **TiV$_5$O$_6$** (0.036 g, 0.045 mmol, 37 %). ^{1}H NMR (500 MHz, MeCN-*d*$_3$): 25.32, 18.32, 16.00, 4.01 ppm. UV-Vis (MeCN, 21 °C) 384 nm (ε = 4185 M^{-1} cm^{-1}), 1095 nm (ε = 530 M^{-1} cm^{-1})). FT-IR (ATR, neat, cm^{-1}), 1134 (O$_t$-CH$_3$) 1008 (O$_b$-CH$_3$), 975 (V=O$_t$). Elemental analysis for TiV$_6$O$_{19}$C$_{13}$H$_{39}$ (MW: 802.01 g mol^{-1}) Calc'd (%): C, 19.47, H 4.90, N, 0.00 Found (%): C 19.68; H 4.77, N, 0.00.

Synthesis of [TiV$_5$O$_5$(OH$_2$)(OCH$_3$)$_{13}$] (**TiV$_5$O$_5$(OH$_2$)**). A 20 mL scintillation vial was charged with **TiV$_5$O$_6$** (0.035 g, 0.044 mmol), H$_2$Phen (0.007 g, 0.040 mmol), and 10 mL of THF. The reaction immediately turned from green to orange. The reaction was stirred for 1 hr to ensure completion, after which the solvent was removed under reduced pressure to yield an orange solid. The solid was washed with 3:1 pentane/diethyl ether mixture (10 mL x 3) until the solution ran clear to remove byproduct (Phen). The solid was extracted in THF and extensively dried to yield **TiV$_5$O$_5$(OH$_2$)** (0.035 g, 0.043 mmol, 79%). ^{1}H NMR (500 MHz, THF-*d*$_8$): δ = 28.19, 27.95, 26.48, 25.23, 16.14, 13.57, 12.86, 6.32, -12.10, -14.38, -17.20, -18.08 ppm. UV-Vis (MeCN, 21 °C): 356 nm (ε = 810 M^{-1} cm^{-1}), 428 nm (ε = 580 M^{-1} cm^{-1}), 524 nm (ε = 240 M^{-1} cm^{-1}), 980 nm (100 M^{-1} cm^{-1}). FT-IR (ATR, neat, cm^{-1}), 1134 (O$_t$-CH$_3$) 1135 (O$_b$-CH$_3$), 976 (V=O$_t$). Elemental analysis for TiV$_5$O$_{19}$C$_{13}$H$_{41}$ ● 0.25 C$_5$H$_{12}$ ● 0.25 MeCN (MW: 832.33 g mol^{-1}) Calc'd (%): C, 21.7, H 5.29, N, 0.43. Found (%): C 21.99; H 5.16, N, 0.41.

General procedure for determining the BDFE(O–H)$_{avg}$ of aquo bound POV through equilibrium experiments.

BDFE(O-H)$_{avg}$ TiV$_5$O$_5$(OH$_2$). In an N$_2$-filled glove box, 100 µL of a 19.9 mM stock solution of **TiV$_5$O$_6$** in THF-*d*$_8$ and 293 µL of THF-*d*$_8$ were added to a J. Young tube. The J. Young tube was capped and removed from the glove box, where a control ^{1}H NMR spectrum was collected. In the glovebox, a separate J. Young tube was loaded with 100 µL of a 19.9 mM stock solution of **TiV$_5$O$_6$** in THF-*d*$_8$ and 293 µL of THF-*d*$_8$, along with 7 µL of a stock solution containing 0.337 M H$_2$Azo in THF-d$_8$. The sample was shaken several times to ensure homogeneity and allowed to sit at 21 °C (room temperature) for 7 days, whereupon the ^{1}H NMR spectrum was collected. This procedure was repeated in quadruplicate. The extent of the reaction was determined through the relative concentrations of reduced H$_2$Azo and oxidized azobenzene (Azo), under the assumption that H-atom transfer occurs solely from the cluster to substrate (*i.e.* for each reduced cluster formed, we assume the oxidation of one molecule of hydrazobenzene). Calculating the BDFE(O–H)$_{avg}$ of the reduced cluster in solution can then be performed through methods adapted from the Mayer group, using Eq. 1.[8]

$$BDFE(E-H)_{adj} = BDFE(E-H)_{avg} - \frac{1.364\ kcal\ mol^{-1}}{n} log\frac{[H_2E]}{[E]} \qquad \text{Eq. 1}$$

where BDFE(*E-H*)$_{adj}$ is the adjusted BDFE of the organic substrate based on where the equilibrium of the system lies, BDFE(*E-H*)$_{avg}$ is the reported BDFE(N–H)$_{avg}$ of H$_2$Azo (60.4 kcal mol^{-1} in THF[9]), *n* is the number of H-atoms transferred to one equivalent of **TiV$_5$O$_6$** (*n* = 2, as **TiV$_5$O$_6$** can accept two H-atom equivalents), and [*H$_2$E*] and [*E*] are the measured concentrations of reduced and oxidized versions of the respective substrate in solution at equilibrium.

General procedure for performing pseudo-first-order reaction kinetics.

Pseudo-first-order reaction conditions were used to obtain approximate rate constant of electron transfer and proton transfer for the reaction between **TiV_5O_6** (0.40 mM) and excess H-atom transfer reagent, H_2Phen (4.0 mM). Samples of cluster stock solutions in THF were loaded in a long-necked quartz cuvette and sealed with a rubber septum before removing from the glovebox. In a 1 mL syringe, a sample of reductant stock solution (102 mM in THF) was measured prior to removal from the glovebox. The UV-Vis-NIR spectrophotometer with temperature controls was set to -50 °C. After equilibrating to -50 °C in the spectrophotometer, data acquisition began, and the reductant solution was forcefully injected to ensure efficient sample mixing. Reaction progression was monitored using absorbances at 1050 and 645 nm over the reaction coordinate. Upon completion of the reaction, the plot of absorbance over time was fit to the following equation by least squares fitting:

$$A_t = A_{inf} + (A_0 - A_{inf})e^{-k_{obs} \times t}$$

where A_t is the absorbance at a given time, t, in seconds, A_{inf} is the absorbance at the end of the reaction (t = infinite), A_0 is the absorbance after reductant injection, and k_{obs} is the observed first order rate constant (s^{-1}). All reactions to determine k_{obsPT} and k_{obsET} were performed in triplicate. Error was determined by calculating the standard deviation between triplicate trials.

Attempts were made to collect k_{obs} for **$V_6O_7^{1-}$** (0.60 mM) under similar conditions to that described above for **TiV_5O_6**. Addition of excess H_2Phen (stock concentration 103 mM) injected using a 1 mL syringe prepared in the glovebox to a solution of **$V_6O_7^{1-}$** at -50 °C in THF. However, little to no reactivity was observed at this temperature. Eyring analysis of **$V_6O_7^{1-}$** reactivity with excess H_2Phen was performed to extrapolate a comparable rate constant at this temperature (see below).

General procedure for determining activation parameters for the reduction of $V_6O_7^{1-}$ via H-atom uptake for comparison to TiV_5O_6.

Eyring analysis of H-atom uptake by **$V_6O_7^{1-}$** was performed by collecting absorbance vs. time data at 1050 nm over temperatures between 5 and 45 °C to extrapolate a rate constant (k_{PCET}) at -50 °C. Reactions were assembled in an analogous fashion to previously run experiments, with constant reductant (10 mM) and cluster (0.60 mM) concentrations ran in triplicate for each temperature. Conversion of k_{obs} to k was done by dividing k_{obs} by the reductant concentration. Plotting ln(k/T) as a function of 1/T (where temperature is converted to K), the linear plot was used to solve for activation parameters using the below equations where R is the gas constant in units of cal (mol^{-1} K^{-1}), k_{Boltz} is Boltzmann's constant, and h_{Planck} is Planck's constant, m is the slope, and b is the y-intercept.

$$ln\frac{k_{PCET}}{T} = m \times \frac{1}{T} + b$$

$$\Delta H^{\ddagger} = m \times -R$$

$$\Delta S^{\ddagger} = R \times [b - \ln\left(\frac{k_{Boltz}}{h_{Planck}}\right)]$$

$$\Delta G^{\ddagger} = \Delta H^{\ddagger} - T\Delta S^{\ddagger}$$

Using the linear relationship between ln(k/T) and 1/T, k at -50 °C for **$V_6O_7^{1-}$** can be extrapolated from the fit of the line. Uncertainties were determined by performing a linear regression function on data from triplicate trials for each condition in Microsoft Excel and calculating a 95% confidence interval. The

reported errors are the first significant figure of the difference between the confidence interval maximum and the values found from the regression. Eyring plots and activation parameters are found in Fig. S14.

Second order rate constants were normalized by a probability factor to account for the number of available V=O sites for PCET to the cluster, $\mathbf{TiV_5O_6}$, n = 4 for the four equatorial vanadyls and $\mathbf{V_6O_7}^{\mathbf{1-}}$, n = 6. Additionally, two H-atom equivalents may be transferred from H_2Phen which are accounted for by dividing the value by two such that the second order rate constants are calculated:

$$k_{PT} = \frac{k_{TiV_5O_6}}{4_{V=O} \times 2_{H-atoms}}$$

$$k_{PCET} = \frac{k_{V_6O_7{}^{1-}}}{6_{V=O} \times 2_{H-atoms}}$$

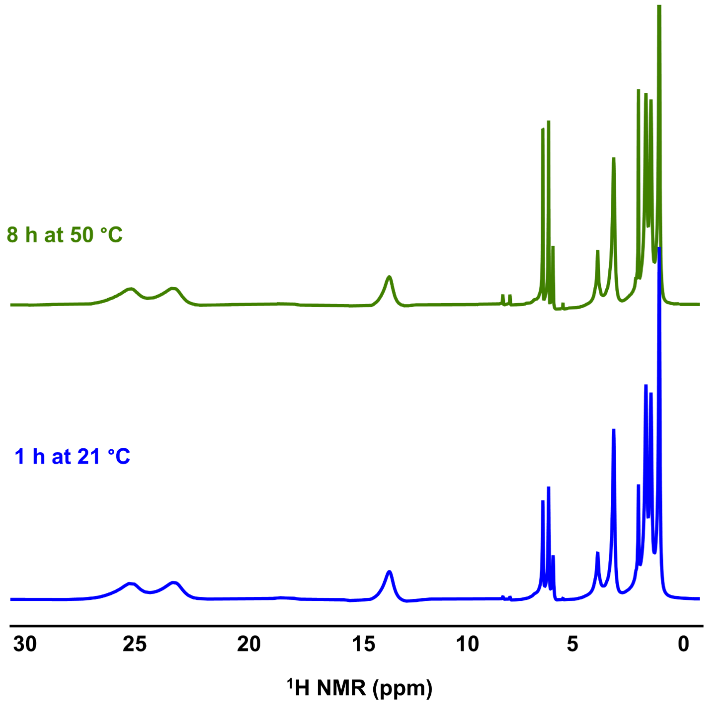

Figure S1. ^{1}H NMR spectra of $[^{n}Bu_4N][TiV_5O_6(OCH_3)_{13}]$ ($\mathbf{TiV_5O_6^{1-}}$) + 1 equiv H_2Phen in MeCN-d_3 at 21 °C (blue) and 8 hr at 50 °C (green).

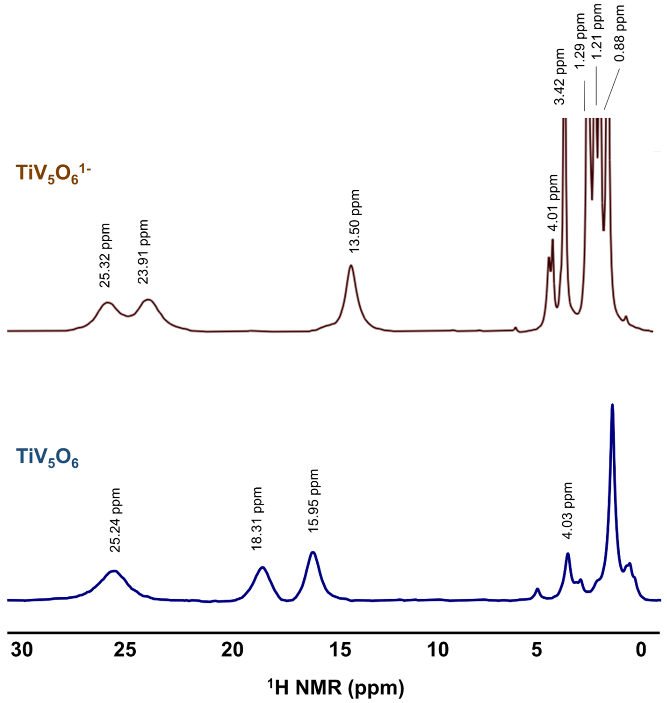

Figure S2. ^1H NMR spectra of $\mathbf{TiV_5O_6^{1-}}$ (brown, top), and $\mathbf{TiV_5O_6}$ (blue, bottom) in MeCN-d_3 at 21 °C.

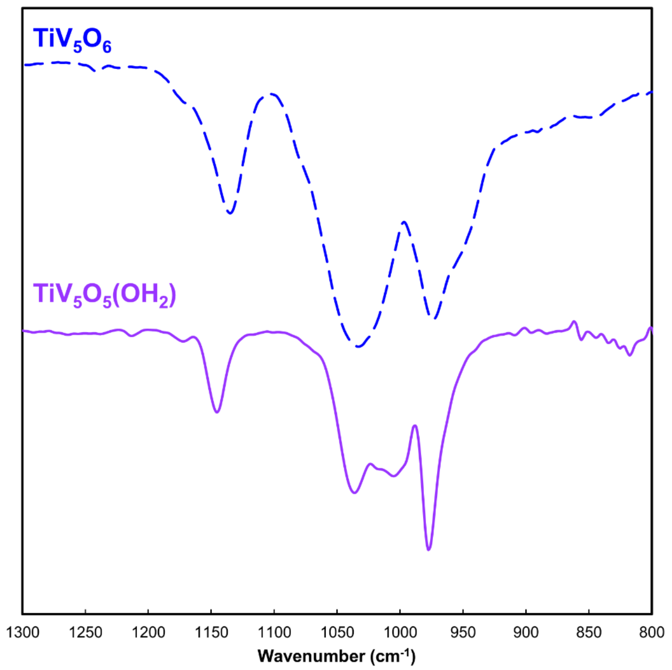

Figure S3. Infrared spectra of $\mathbf{TiV_5O_6}$ (blue, dashed line) and $\mathbf{TiV_5O_5(OH_2)}$ (purple, solid line), neat.

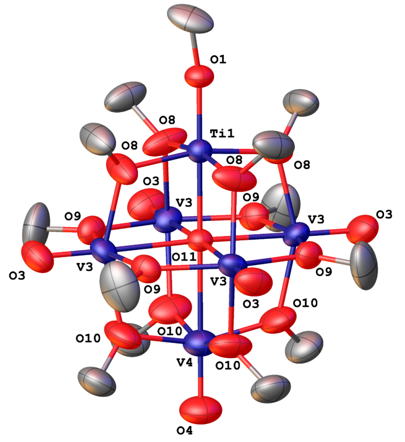

Figure S4. Crystal Structure of $TiV_5O_6(OCH_3)_{13}$ (**TiV_5O_6**).

Table S1. Crystal data and structure refinement for [TiV_5O_6] (**CCDC 2304273**)

Identification code	[TiV_5O_6]	
Empirical formula	C_{13} H_{39} O_{19} Ti V_5	
Formula weight	802.04	
Temperature	100.00(10) K	
Wavelength	1.54184 Å	
Crystal system	tetragonal	
Space group	$P42_12$	
Unit cell dimensions	*a* = 20.08710(10) Å	a = 90°
	b = 20.08710(10) Å	b = 90°
	c = 14.52010(10) Å	g = 90°
Volume	5858.74(7) Å^3	
Z	8	
Reflections collected	50716	
Independent reflections	6376 [*R*(int) = 0.0621]	
Completeness to theta = 74.504°	99.9%	
Goodness-of-fit on F^2	1.049	
Final *R* indices [*I*>2sigma(*I*)]	*R*1 = 0.0480, *wR*2 = 0.1317	
Largest diff. peak and hole	0.844 and -0.397 e.Å^{-3}	

Table S2. Comparison of selected structural parameters of $\mathbf{TiV_5O_6}$ and $\mathbf{TiV_5O_6^{1-}}$.[7]

**Bond**	$\mathbf{TiV_5O_6}$	$\mathbf{TiV_5O_6^{1-}}$
Ti-O_m	1.771(7) Å	1.783(5) Å
Ti-O_m-C	164.9(6)°	173.7(7)°
Ti-O_b (range)	1.962(4) – 1.984(4) Å	1.958(5) – 1.980(5) Å
Ti-O_c	2.030(5) Å	2.042(5) Å
V_e-O_c (range)	2.3343(11) – 2.3349(12) Å	2.334(4) – 2.352(4) Å
V_e=O_t (range)	1.592(5) – 1.596(5) Å	1.603(5) – 1.605(4) Å
V_a-O_c	2.500(5) Å	2.4928(45) Å
V_a=O_t	1.583(6) Å	1.598(5) Å

Table S3. Bond valence sum calculations for vanadium atoms in $\mathbf{TiV_5O_6}$.

**Bond**	**V5 (V_e)**	**V6 (V_e)**	**V7 (V_a)**
V(III)	3.971	4.072	4.192
V(IV)	**4.066**	**4.169**	4.292
V(V)	4.333	4.443	**4.569**

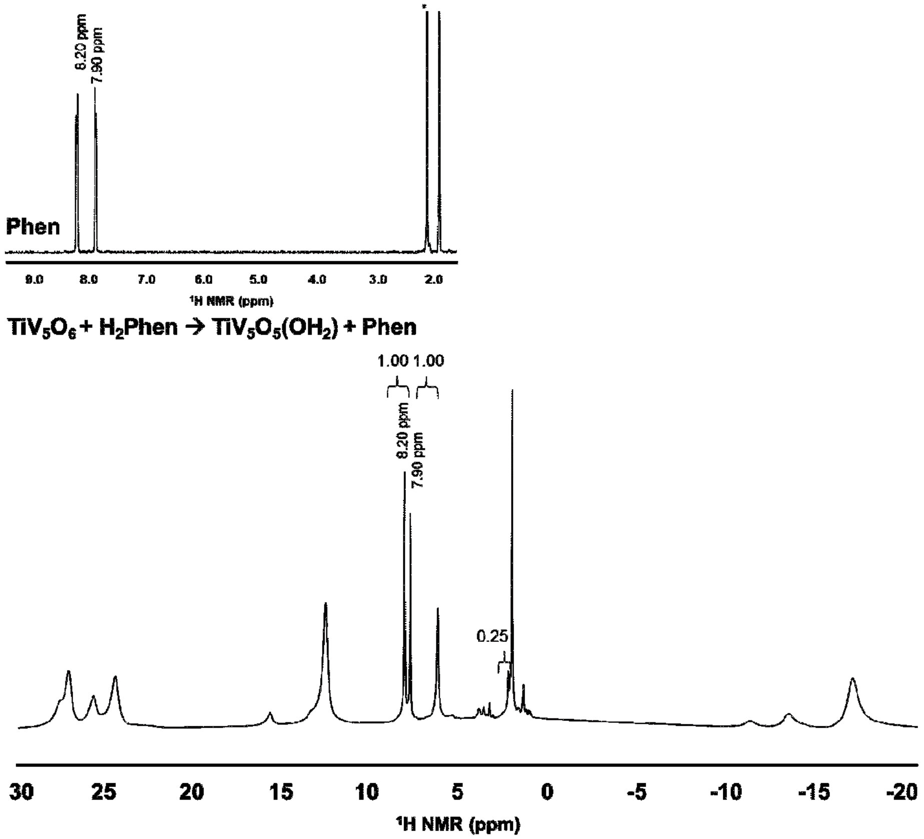

Figure S5. Crude ^{1}H NMR of $\mathbf{TiV_5O_6}$ + 1 equiv H_2Phen in MeCN-d_3 at 21 °C after 2 hr to show the formation of $\mathbf{TiV_5O_5(OH_2)}$ and Phen (8.20, 7.90 ppm). Inset shows the spectrum of Phen in MeCN-d_3 at room temperature (21 °C).**

*Indicates water, spectrum was not taken using anhydrous deuterated solvent.

** The amount of water generated is less than that observed in the case of the homometallic clusters (~0.25 equiv vs expected 1.00 equiv),[10] suggesting that equilibrium favors coordination of water to the surface of the assembly, instead of dissociation of OH_2 in favor of a CH_3CN solvent molecule. Integrations are noted in the figure above the peaks by brackets.

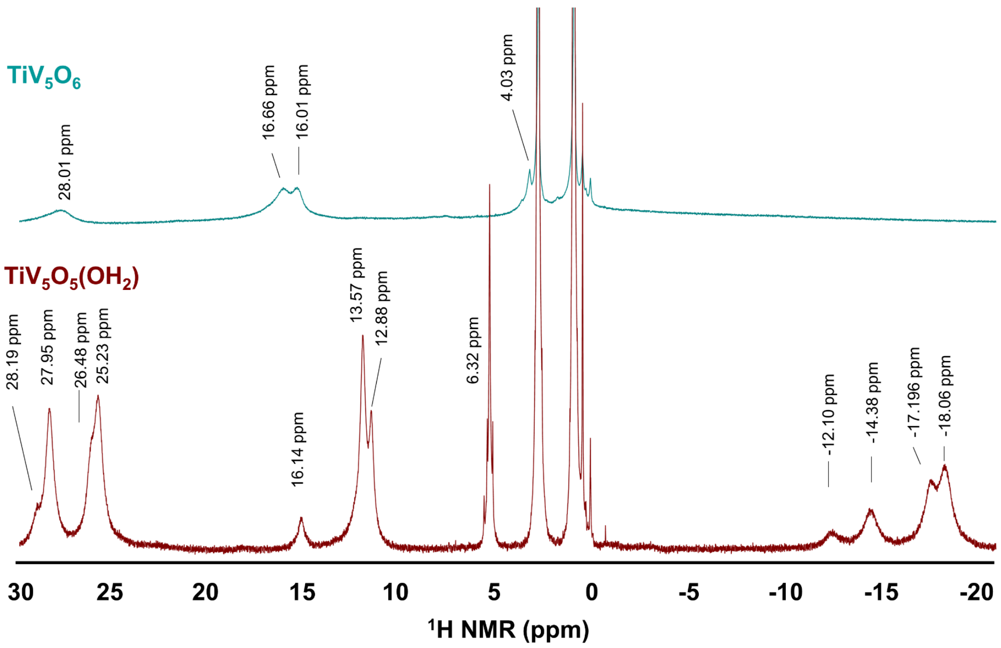

Figure S6. ^{1}H NMR spectra of **TiV_5O_6** (light blue, top) and **$TiV_5O_5(OH_2)$** (brown-red, bottom) collected in THF-d_8 at room temperature (21 °C).

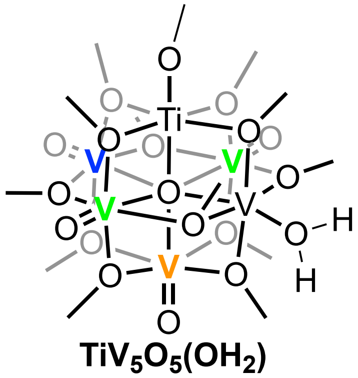

Figure S7. Illustration of **$TiV_5O_5(OH_2)$** shown to delineate chemically unique V(IV) centers. Chemically unique V(IV) ions are shown in blue, green, and orange.

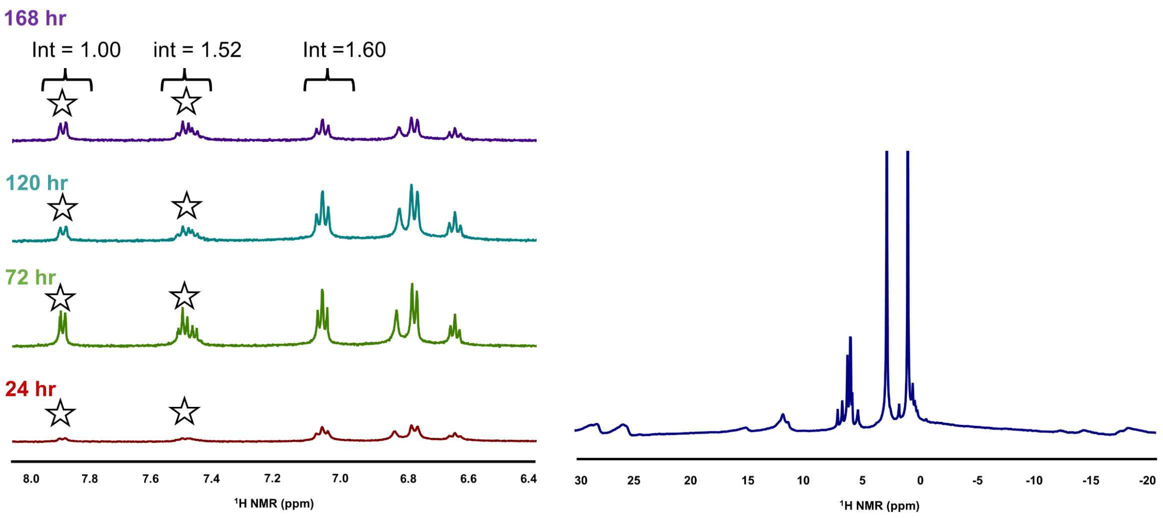

Figure S8. Diamagnetic ^{1}H NMR of **TiV_5O_6** + 1 equiv H_2Azo in THF-d_8 over time at 21 °C, until equilibrium is reached (left, trial C). Paramagnetic ^{1}H NMR of **TiV_5O_6** + 1 equiv H_2Azo in THF-d_8 21 °C (room temperature), after equilibrium was reached (right). Stars indicate the formation of Azo, integration is noted via bracket at 168 hr.

Table S4. BDFE(O-H)$_{adj}$ calculated from equilibrium reactions of **TiV_5O_6** with H_2Azo after 7 days.

	**H_2Azo**[b]		**Azo**					
	7.06 ppm (4 H)		7.92 ppm (4 H)		7.52 ppm (6 H)			
Trial	Integral	Relative Conc.[a]	Integral	Relative Conc.[a]	Integral	Relative Conc.[a]	$[H_2E]/[E]$[c]	$BDFE_{adj}$
A	3.02	0.76	1.0	0.25	1.53	0.26	8.21	59.77
B	2.46	0.62	1.0	0.25	1.54	0.26	4.70	59.94
C	1.6	0.4	1.0	0.25	1.52	0.25	4.30	59.96
D	4.0	1.0	1.0	0.25	1.56	0.26	1.52	60.27
							BDFE(avg)	60.0
							STDV	0.1

[a] Relative concentration of either H_2Azo or Azo was determined by normalizing the integral to the number of protons by dividing it by the number of known protons in a given peak.

[b] Integration of the N-H resonance of H_2Azo was omitted in this study due to potential broadening of the resonance by H-bonding with **TiV_5O_6**.

[c] $[H_2E]$ is the average relative concentration of H_2Azo, [E] is the average relative concentration of Azo.

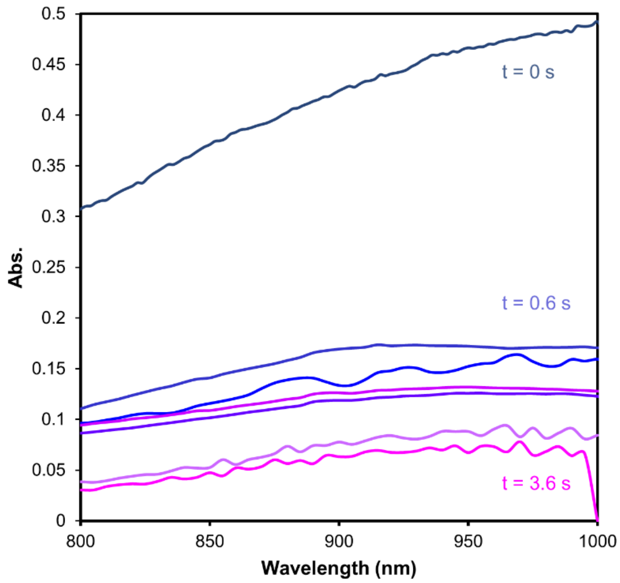

Figure S9. Electronic absorption spectra (EAS) of $\mathbf{TiV_5O_6}$ (0.75 mM) + 10 equiv H_2Phen (7.5 mM) in MeCN over time at -35 °C. Reaction is ~60% complete by the time the first scan is taken, complicating kinetic analysis under pseudo-first order conditions.

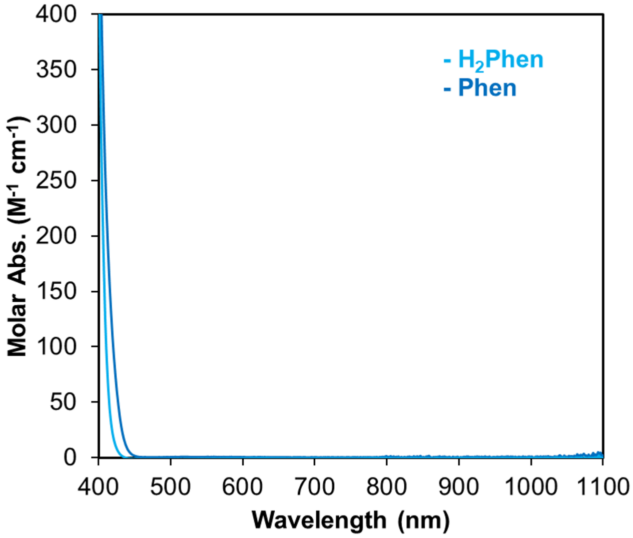

Figure S10. EAS of H_2Phen (0.50 mM), light blue, and Phen (0.33 mM), dark blue, collected in MeCN at room temperature (21 °C).

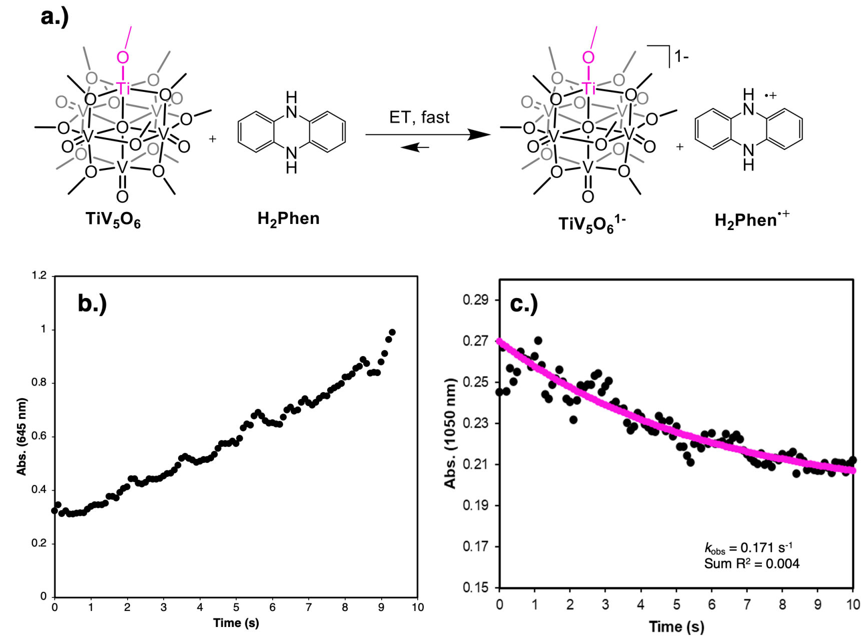

Figure S11. Determining a lower bound of k_{obsET} for the electron transfer to **TiV_5O_6** and formation of $H_2Phen^{\cdot+}$. (a) Reaction scheme for ET to 0.4 mM **TiV_5O_6** from 4.0 mM H_2Phen in THF at – 50 °C (b) Kinetic trace at 645 nm showing the growth of $H_2Phen^{\cdot+}$ (data indicated by black circles). (c) Kinetic trace at 1050 nm showing the loss of **TiV_5O_6** (data indicated by black circles, fit indicated by pink line, $k_{obsET} = 0.171\ s^{-1}$).

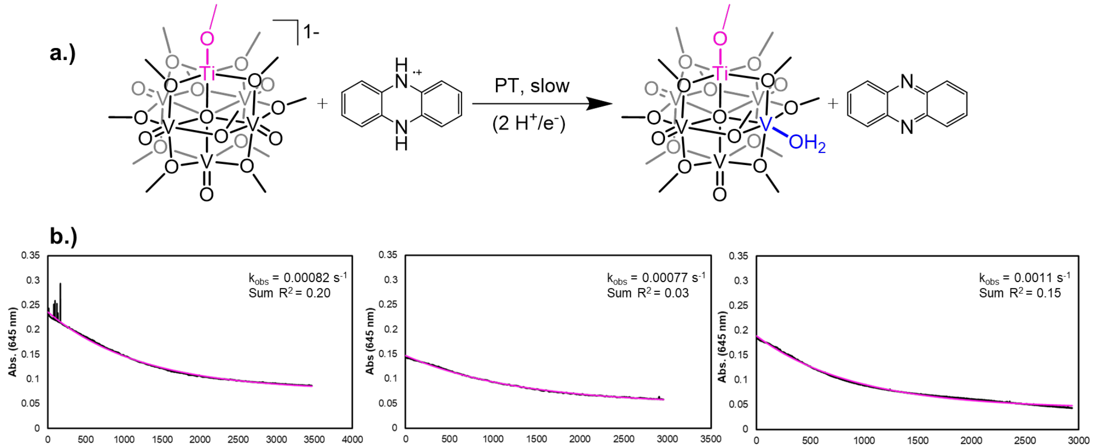

Figure S12. Determining a lower bound of k_{obsPT} for the proton transfer to **$TiV_5O_6^{1-}$** and loss of $H_2Phen^{\cdot+}$, where t_0 is corrected to start of loss of $H_2Phen^{\cdot+}$. (a) Reaction scheme for rate limiting PT from initial reaction of 0.4 mM **TiV_5O_6** from 4.0 mM H_2Phen in THF at – 50 °C (b) Kinetic trace at 645 nm showing the loss of $H_2Phen^{\cdot+}$ (data indicated by black circles, fit indicated by pink line). Repeated in triplicate, where the average value is reported $k_{obsPT} = 0.00090\ s^{-1}$ with error reported from the standard deviation of $0.000014\ s^{-1}$. A second order rate constant is estimated by dividing the pseudo-first order rate constant by the concentration of reductant and the probability factor to get $k_{PT} = 0.028 \pm 0.005\ M^{-1}\ s^{-1}$.

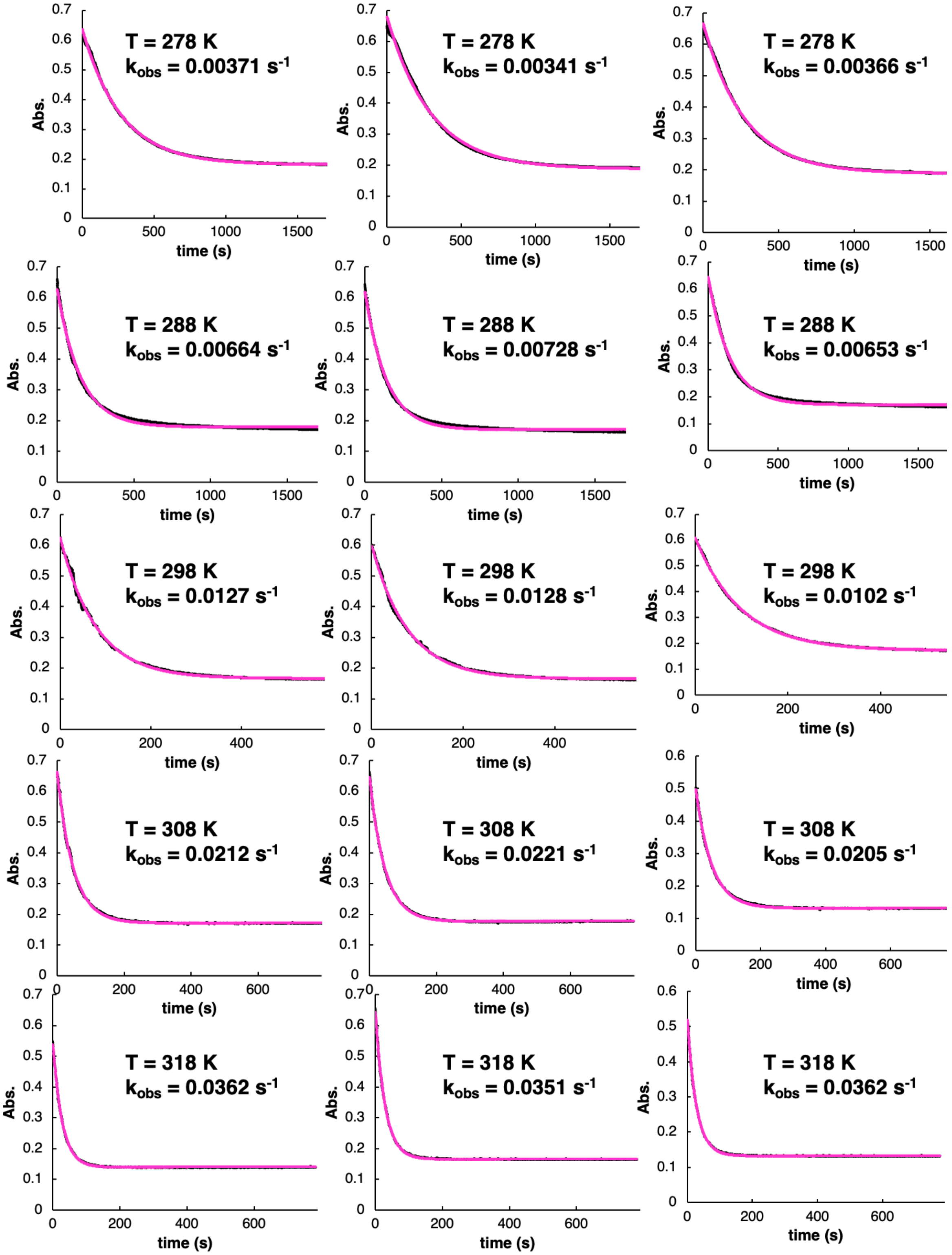

Figure S13. Kinetic traces for the reaction between 0.6 mM $\mathbf{V_6O_7}^{1-}$ + 10 mM H_2Phen in THF at 1050 nm at variable temperatures (T = 278, 288, 298, 308, 318 K) repeated in triplicate. Data is indicated by black line and fit is indicated by pink line. k_{obs} is noted for each trial.

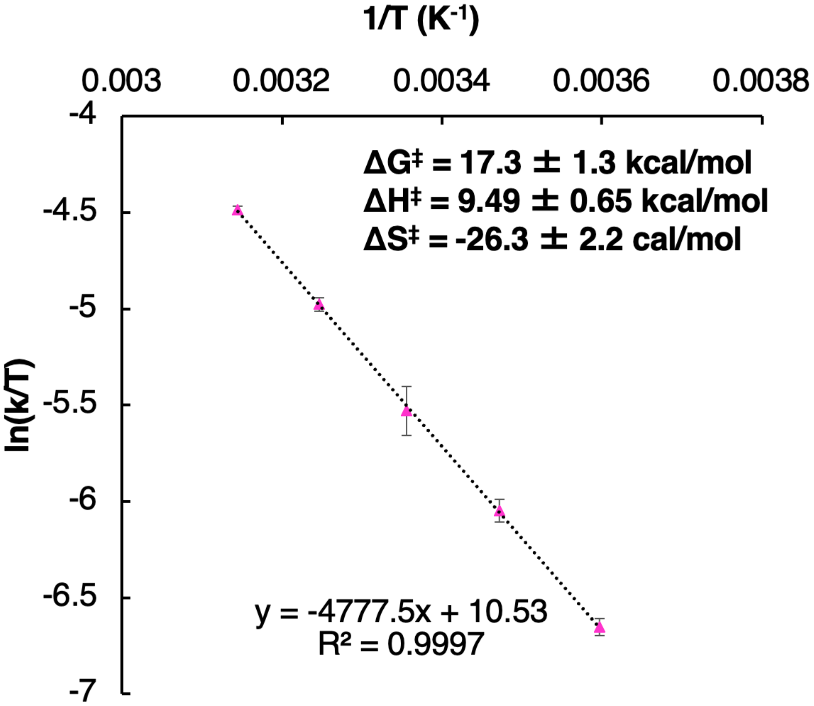

Figure S14. Eyring parameters for the reaction of 0.6 mM $\mathbf{V_6O_7}^{1-}$ + 10 mM H_2Phen in THF. *k* is extrapolated from the plot to determine the second order rate constant at -50 °C. $\Delta G^{\ddagger}$ is calculated at 298 K.

References.

1. Lee, J.; Shizu, K.; Tanaka, H.; Nakanotani, H.; Yasuda, T.; Kaji, H.; Adachi, C. Controlled emission colors and singlet–triplet energy gaps of dihydrophenazine-based thermally activated delayed fluorescence emitters. *J. Mater. Chem. C* **2015**, *3*, 2175-2181.
2. Spandl, J.; Daniel, C.; Brüdgam, I.; Hartl, H. Synthesis and Structural Characterization of Redox-Active Dodecamethoxoheptaoxohexavanadium Clusters. *Angew. Chemie Int. Ed.* **2003**, *42*, 1163-1166.
3. C. Daniel and H. Hartl, *J. Am. Chem. Soc.*, **2009**, 131, 5101-5114
4. CrysAlisPro. *Rigaku Corporation* **2023**.
5. Sheldrick, G. M. SHELXT. *Acta. Crystallogr.* **2015**, *version 2018/2;*, 3-8.
6. Sheldrick, G. M. SHELXL. *Acta. Crystallogr.* **2015**, *version 2019/2*, 3-8.
7. VanGelder, L. E.; Brennessel, W. W.; Matson, E. M. Tuning the redox profiles of polyoxovanadate-alkoxide clusters via heterometal installation: toward designer redox Reagents. *Dalton Trans.* **2018**, *47*, 3698-3704.
8. Agarwal, R. G.; Kim, H.-J.; Mayer, J. M. Nanoparticle O–H Bond Dissociation Free Energies from Equilibrium Measurements of Cerium Oxide Colloids. *J. Am. Chem. Soc.* **2021**, *143*, 2896-2907.
9. Cooney, S. E.; Fertig, A. A.; Buisch, M. R.; Brennessel, W. W.; Matson, E. M. Coordination-induced bond weakening of water at the surface of an oxygen-deficient polyoxovanadate cluster. *Chem. Sci.* **2022**, *13*, 12726-12737.
10. Schreiber, E.; Fertig, A.A.; Brennessel, W.W.; Matson, E.M. Oxygen-atom defect formation in polyoxovanadate clusters via proton-coupled electron transfer. *J. Am. Chem. Soc.* **2022**, *144*, 5029-5041.
